# Violence and post-traumatic stress disorder in Sao Paulo and Rio de Janeiro, Brazil: the protocol for an epidemiological and genetic survey

**DOI:** 10.1186/1471-244X-9-34

**Published:** 2009-06-07

**Authors:** Sérgio Baxter Andreoli, Wagner Silva Ribeiro, Maria Ines Quintana, Camila Guindalini, Gerome Breen, Sergio Luis Blay, Evandro SF Coutinho, Trudy Harpham, Miguel Roberto Jorge, Diogo Rizzato Lara, Tais S Moriyama, Lucas C Quarantini, Ary Gadelha, Liliane Maria Pereira Vilete, Mary SL Yeh, Martin Prince, Ivan Figueira, Rodrigo A Bressan, Marcelo F Mello, Michael E Dewey, Cleusa P Ferri, Jair de Jesus Mari

**Affiliations:** 1 Department of Psychiatry, Universidade Federal de São Paulo, São Paulo, Brazil; 2 MRC Social, Institute of Psychiatry, King's College, London, UK; 3 Escola Nacional de Saúde Pública (ENSP – FIOCRUZ), Rio de Janeiro, Brazil; 4 London South Bank University, London, UK; 5 London School of Hygiene and Tropical Medicine, London, UK; 6 Faculdade de Biociências da PUCRS, Porto Alegre, Brazil; 7 Centre for Public Mental Health, Health Services and Population Research Department, Institute of Psychiatry, King's College, University of London, London, UK; 8 Institute of Psychiatry, Universidade Federal do Rio de Janeiro (IPUB – UFRJ), Rio de Janeiro, Brazil

## Abstract

**Background:**

violence is a public health major concern, and it is associated with post-traumatic stress disorder and other psychiatric outcomes. Brazil is one of the most violent countries in the world, and has an extreme social inequality. Research on the association between violence and mental health may support public health policy and thus reduce the burden of disease attributable to violence. The main objectives of this project were: to study the association between violence and mental disorders in the Brazilian population; to estimate the prevalence rates of exposure to violence, post-traumatic stress disorder, common metal disorder, and alcohol hazardous use and dependence: and to identify contextual and individual factors, including genetic factors, associated with the outcomes.

**Methods/design:**

one phase cross-sectional survey carried out in Sao Paulo and Rio de Janeiro, Brazil. A multistage probability to size sampling scheme was performed in order to select the participants (3000 and 1500 respectively). The cities were stratified according to homicide rates, and in Sao Paulo the three most violent strata were oversampled. The measurements included exposure to traumatic events, psychiatric diagnoses (CIDI 2.1), contextual (homicide rates and social indicators), and individual factors, such as demographics, social capital, resilience, help seeking behaviours. The interviews were carried between June/2007 February/2008, by a team of lay interviewers. The statistical analyses will be weight-adjusted in order to take account of the design effects. Standardization will be used in order to compare the results between the two centres. Whole genome association analysis will be performed on the 1 million SNP (single nucleotide polymorphism) arrays, and additional association analysis will be performed on additional phenotypes. The Ethical Committee of the Federal University of Sao Paulo approved the study, and participants who matched diagnostic criteria have been offered a referral to outpatient clinics at the Federal University of Sao Paulo and Federal University of Rio de Janeiro.

## Background

Over the past decades, violence became a major public health concern worldwide. In 1993, the Directing Council of the Pan American Health Organization declared the prevention of violence to be a public heath priority, and three years later the World Health Assembly proposed a similar resolution [1]. The World Report on Violence and Health [2] estimates that more than 1.6 million people worldwide died in 2000 as a result of violence. According to the report, nearly half of these deaths were suicides, almost one-third were homicides, and one-fifth were war-related deaths. More than 90% of these deaths occurred in low and middle-income countries (LAMIC). The African and Latin American regions have nearly three times more homicides than suicides while in European and South East Asian regions suicide rates are twice as high than the homicide rates [2,3]. The average rate of homicides in the Americas between 2000 and 2005 was the highest worldwide (17.8 per 100,000 inhabitants), and in Brazil specifically the average homicide rate during the same period ranked fourth highest in the Americas (31.0 per 100,000 inhabitants) [4]. Moreover, 82% of all homicides in the region occurred in Brazil, Colombia and Mexico [5].

It is well known that violence causes much more injuries than deaths, and that it is accompanied by social and psychological impacts. Exposure to violence has been associated to several mental health problems, including suicide, substance misuse, depression, and post-traumatic stress disorder [3,6]. For instance, women reporting intimate partner violence are two to three times more likely to be depressed than women without history of victimisation by violence [6]. Moreover, post-traumatic stress disorder is the most frequent psychiatric outcome of exposure to violence. Epidemiological surveys in the United States' general population have shown that 15% to 24% of those exposed to violence will develop PTSD [7]. In the US, the lifetime DSM-IV prevalence of PTSD was found to be around 6.8% [8], while in low income countries where people have experienced war, conflict or mass violence the rates were found to be much higher (15.8% in Ethiopia, 17.8% in Gaza Strip, 28.4% in Cambodia, and 37.4% in Algeria [9].

Most people exposed to traumatic events actually do not develop PTSD, what might be explained by differences in factors related to vulnerability and resilience [10]. Just as most other mental disorders, PTSD seems to be a result of a complex equation in which both individual and environmental factors play an important role either by increasing the vulnerability for developing the disorder, or by helping to cope with the deleterious effects of the traumatic experience. Gender differences have been found in several population surveys on PTSD. These studies have shown that women are at greater risk of developing PTSD after exposed to traumatic events. It has been hypothesized that women are at greater risk either due their greater physiological reactivity, or due the fact that women are more likely than men to experience the most potentially traumatic events, such as interpersonal violence. Cultural and social theories have also been proposed in order to explain the women's greater vulnerability. According to those theories, men may suppress symptoms, since the cognitions related to trauma, such as fear and helplessness are dissonant with men's self-concepts. Moreover, more women than men – mainly those living in low and middle-income countries – tend to be exposed to socioeconomic stressors, such as poverty, discrimination and oppression, which can reduce the capacity to cope with the adversity [7,11-18]. Socioeconomic disadvantages have been described as one of the most important emotional stressors, and several studies have found poverty and socioeconomic deprivation to be associated to common mental disorders [19,20]. Studies on PTSD have found similar results. Psychiatric history is another factor frequently associated to PTSD. It has been found that up to 87.5% of people diagnosed with PTSD have at least one additional diagnosis. Anxiety disorders, depressive disorders and substance related disorders have been found to be both predictors, comorbid conditions, or secondary to PTSD [15]. Finally, there is evidence that the cognitive, emotional and behavioural reactions during the traumatic event play an important role in the subsequent development of PTSD, in the severity of the symptoms, and response to treatment [18], since they may interfere with the processing of traumatic memories [21].

Several studies have examined the genetic contribution on the aetiology of PTSD. Sack et al. [22] found the risk for PTSD to be significantly higher in first degree relatives of patients. A large study, that included 4,042 monozygotic and dizygotic male twin pairs of Vietnam War veterans, found inheritance to have a substantial influence on liability for all symptoms of PTSD [23]. Although it seems to be clear that genetics accounts for part of the familial cluster pattern in PTSD, no monogenic inheritance has been found and a more complex pattern of inheritance has been suggested [24]. This suggests that there are multiple genetic risk loci for PTSD each partially contributing to the risk for developing the disorder, and current thinking in genetic would suggest that their exact effects may be dependent on specific gene-environment interactions. Some studies have linked the dopaminergic and serotoninergic systems to individual response to trauma and it has been suggested that PTSD is associated with a functional deficit in dopaminergic system that compromises the ability to deal with the traumatic event [25]. Kilpatrick et al. (2007) [26] found variation in 5-HTTLRP moderated risk of developing PTSD in adults exposed to the 2004 Florida Hurricanes. Furthermore, the presence of A1+ allele (A1A2, A1A1 genotypes) of dopamine D2 receptor genes has been associated with a higher incidence of PTSD [27] and Segman [28] has found an association of the allele 9 of the dopamine transporter gene (DAT1) polymorphism and the development of PTSD. Other neurotransmitters systems have also been implicated in tolerance to stress and response to violence. Functional polymorphism of monoamine-oxidase has been found to be associated with anti-social conducts after exposure to maltreatment in childhood [29] and polymorphism of the serotonin transporter gene (5-HTTLPR) has been found to be associated with response to stress; the short allelic variant is associated with decreased amygdala neuronal activity in response to danger when compared to the long variant [30-32]. In spite of these evidences linking genetic variances to tolerance to stress and PTSD, genetic studies on PTSD are surprisingly rare and no candidate gene has yet been identified.

In summary, the literature shows that urban populations worldwide are highly exposed to traumatic events, and that the prevalence of mental disorders relates to this exposure is expressive. However, few studies were carried out in the developing world, whose population is exposed to the highest levels of violence and social disadvantages. The available data shows that prevalence rates of PTSD tend to be much higher in low and middle-income countries than in the US and Europe. Moreover, the evidence on risk factors for trauma-related mental disorders remains inconclusive, and, to date, there are no population studies on resilience factors related to the traumatic experience. Since the exposure to traumatic events represents a modifiable risk factor both at population and individual levels [33], epidemiological studies on traumatic events and mental disorders may raise awareness of the burden of disease resulting of the deleterious effects of violence on the mental health.

The primary aim of this survey is to study the exposure to urban violence and the effects of this exposure on the mental health in the two Brazilian major urban areas. Sao Paulo and Rio de Janeiro were chosen as the settings for this study due to their high levels of violence and social inequality. The association between violence and inequality in the two cities may provide a huge variability in terms of victimization, outcomes, and correlated factors. This variability is expected to allow the identification of potential vulnerability and resilience factors.

Based on the characteristics of the of Sao Paulo and Rio de Janeiro, it is hypothesized that the prevalence rates of exposure to traumatic events will be expressive, and that most of the population will report at least one life-threatening experience over the course of their lives. The prevalence of PTSD is expected to be considerably higher in the two cities than that reported in developed countries. Moreover, the prevalence rates should vary across the cities, being much higher in the most violent regions. Both the most severe types of violence and the highest rates of mental disorders are expected to be associated to poorest social indicators both at individual and community levels. Finally, it is expected that higher social capital and resilience scores will be associated to lower rates of mental disorders even among those exposed to the most extreme forms of violence.

The study's aims are:

1) To estimate the prevalence of exposure to traumatic life events, particularly to violence, in the populations of Sao Paulo and Rio de Janeiro, Brazil.

2) To estimate the prevalence of post-traumatic stress disorders, common mental Disorders (CMD), and alcohol hazardous use and dependence.

3) To study the association between traumatic events and PTSD, common mental disorders and hazardous alcohol use and dependence.

4) To identify potential vulnerability and resilience factors at both individual and community level related to exposure to violence and the development of CMD;

5) To study the influence of contextual variables, such as district homicide rates and district Gini index, on the prevalence of PTSD, common mental disorders and alcohol hazardous use and dependence.

6) To identify genetic factors which predispose to PTSD:

a. Using the 1 million SNP (single nucleotide polymorphism) arrays to conduct a GWAS for Common Mental Disorders (CMDs) with symptoms counts of General Anxiety Disorder and PTSD as the primary phenotypes and other disorders in following secondary analyses.

b. Using admixture mapping approaches for PTSD and other Common Mental Disorder traits;

## Methods/design

### Study design

A one-phase population based cross-sectional survey has been conducted in the cities of Sao Paulo and Rio de Janeiro. Sao Paulo and Rio de Janeiro are the two biggest Brazilian cities.

### Settings

Located in the southwest region of the country, Sao Paulo (population of 11 million inhabitants) is the biggest and richest Brazilian city. It is the most important Brazilian industrial and, commercial and financial centre, where the most important companies are headquartered. In 2006, the city's gross domestic product (GDP) was estimated to be around 124 billion US dollars, and the GDP per capta was of nearly 11 thousand US dollars/year, and in 2003, the GINI index was of 0.45. The current unemployment rate in the city is of 8.6%. With 6 million inhabitants, Rio de Janeiro is the second biggest city in Brazil. Its economy is predominantly based on services, and its GDP was of around 56 billion US dollars in 2006, the GDP per capta being equivalent to 9.1 thousand US dollar/year. The GINI index in 2003 was of 0.48, and the current unemployment rate is estimated to be 6.3%. Both Sao Paulo and Rio de Janeiro are among the most violent cities in the country. In 2003, the average homicide rates in the cities were of 47.13 and 44.3 homicides per 100,000 inhabitants respectively, while in the country as a whole it was of 28.6. Just as the social indicators, homicide rates vary considerably across the cities, being: in 2003, they varied from 2.90 to 88.20 across the 96 administrative districts in Sao Paulo, and from 0 to 91.77 in 33 administrative regions in Rio de Janeiro [4,34,35].

### Sampling procedure

In order to draw representative samples of the population aged 15 to 75 years, a multistage probability to size sampling scheme was performed. In the first stage, the different areas within the two cities were ranked according to their homicide rates, and then grouped into seven strata (1 = less than 10 homicides/100,000 inhabitants; 2 = 10.01 to 20; 3 = 20.01 to 30; 4 = 30.01 to 40; 5 = 40.01 to 50; 5 = 50.01 to 60; and 6 = more than 60 homicides/100,000 inhabitants). In the second stage, all the census sectors within each stratus were mapped. A number of census sectors was randomly selected within each stratus. The number of census sectors varied form 4 to 18 according to the population size within each stratus. In the third stage, 43 households (Sao Paulo) or 30 households (Rio de Janeiro) where randomly selected within each census sector on the base of odd random numbers. In each selected household all residents aged 15 to 75 yeas were enumerated, and one of them was randomly selected based on the Kish's method.

Precision calculations indicated that a sample size of around 850 interviews would allow estimation of lifetime prevalence of PTSD of 10%, within a 95% confidence interval. Due an expected refusal rate of 20%, and in order to identify current PTSD cases to be referred to a case-control study and to a clinical trial, the sample size was established to be of 3,000 interviews in Sao Paulo, and 1,500 interviews in Rio de Janeiro. In Sao Paulo, the three most violent strata were oversampled.

### Measurements

The interview included a number of fully structured questionnaires and scales which have been widely applied in epidemiological surveys. Most of them had been previously translated into Portuguese and validated to the Brazilian cultural context. Those who had not been translated yet were carefully translated into Portuguese by the authors of the study. All participants answered to the full assessment, which lasted approximately 1.5 – 2.5 hours. The complete assessment included:

*Mental health: *psychiatric diagnoses assessed in the study are: a) post-traumatic stress disorder (PTSD); b) phobic and anxiety disorders; c) depressive disorders; d) alcohol hazardous use and dependence. All diagnostics were assessed through the version 2.1 of Composite International Diagnostic Interview (CIDI 2.1), which is a standardized, fully structured interview for the diagnosis and classification of mental disorder according to the International Classification of Diseases, 10^th ^edition (IDC-10), and the Diagnostic and Statistical Manual of the American Psychiatric Association, 4^th ^edition (DSM-IV). The Brazilian version of CIDI 2.1 was previously validated. Sensitivity and specificity for depressive disorder (82.5% and 92.8%), phobic-anxiety disorders (80.6% and 93.5%) and alcohol hazardous use and dependence (79.5% and 97.3%) were found to be satisfactory [36]. When compared to the Structured Clinical Interview (SCID), the CIDI 2.1 PTSD section had sensitivity of 82.4% and specificity of 84.8% for the ICD-10 PTSD diagnostic criteria, and sensitivity of 51.5% and specificity of 94.1% for the DSM-IV criteria (Quintana, MI; Ribeiro, WS; Mari, JJ; Jorge, MR; Andreoli, SB: The Validity of the Post-traumatic Stress Disorder section of the Composite International Diagnostic Interview – CIDI 2.1, submitted).

*Exposure to traumatic events *was assessed through the list of traumatic events of the CIDI 2.1. The list was adapted, and 22 new events were added to the 11 original events. Information on the frequency, intensity, first exposure, and last exposure were also obtained.

*Community level exposure *includes homicide rates Gini index and other social indicators, such as human development index (HDI), unemployment rates and literacy levels. These measures have been estimated for each district using the available data from the Sao Paulo State Data Analysis System Foundation (SEADE) [35] and the Brazilian Institute of Geography and Statistics (IBGE) [34].

#### Socio-demographics

The socio-demographic assessment included gender, age, marital status, number of children, education, employment status, income (individual and family), religion affiliation and practice; migration history.

*Social capital *was assessed through the Short Social Capital Assessment Tool (SASCAT). The SASCAT is a shortened version of the Adapted Social Capital Assessment Tool (A-SCAT), and is specially designed to measure cognitive and structural social capital in low-income countries. The scale comprises questions which measure three aspects of structural social capital (membership of groups, support from individuals and groups in the community and involvement in citizenship activities), as well as cognitive social capital, which comprises trust, social harmony, perceiving fairness, and sense of belonging [37]. The SASCAT was carefully translated and adapted by the authors of the current survey.

*Help seeking behaviour *specific questions were added to the questionnaire in order to assess the seeking for both professional and community help for mental health problems.

*Subjective well-being: *subjective well-being has been reported as a composite measure of independent feeling about a variety of life concerns, in addition to an overall feeling about life in positive and in negative terms. General well-being appears to be stable over time to an extent that its positive and negative affects can be considered as personality traits. Subjective well-being was assessed through six questions from the Subjective Well-being Inventory (SUBI). These questions assess three different domains of the subjective well-being: a) general well-being positive affect; b) expectation-achievement congruence; and c) transcendence [38].

*Resilience: *resilience can be defined as the social and psychological processes related to the individual health development even when exposed to adverse experiences. It comprises different individual and community factors which can be act as protective factors against the adversity: a) personality traits, such as self-esteem, flexibility and ability to deal with conflict; b) family cohesion; c) availability of external support, especially from peers and community. This construct was assessed through the Brazilian version of the Wagnild & Young's Resilience Scale, which has already been cross-culturally adapted [39].

*Positive and negative affects: *positive affect (PA) and negative affect (NA) reflect individual differences in positive and negative reactivity. It has been demonstrated that PA and NA correspond to the dominant personality factors of extroversion and anxiety/neuroticism, respectively. PA and NA are hypothesised to be potential risk (NA) or protective (PA) factors related to the development of mental disorders. The Positive and Negative Affect Schedule was applied in order to assess these factors [40,41].

*Life style*: tobacco was assessed through three questions about current use (yes/no), frequency, and quantity; illicit psychoactive substances use was assessed through questions about use (more than five times) during the last 12 months (no/yes), drugs' names, and use during the last month (no/yes).

*Psychotropic medications use *was assessed through questions about taking medications for convulsions, or psychological/psychiatric problems during the last 12 months; medications' names; professional/person by whom they were prescribed; how/where the medications were obtained; and use during the last month.

### Trauma-related reaction

*Peritraumatic dissociation *is a set of subjective experiences which includes alterations in the perception of time, place, and self during and immediately after trauma exposure [42]. Evidence has shown a direct correlation between peritraumatic dissociative symptoms and the development of PTSD. These symptoms were assessed through the Brazilian version of the Peritraumatic Dissociative Experiences Questionnaire (PDEQ) [43].

*Peritraumatic tonic immobility (PTI) *is a set of involuntary motor reactions characterized by freezing or immobility that occur in the face of life-threatening overwhelming situations. PTI has been found to be associated to pos-traumatic stress symptoms [44,45]. It was assessed through the Tonic Immobility Scale, which has been previously translated into Brazilian Portuguese, and has been used in research and clinical settings.

*Peritraumatic panic attacks *have been found to modulate the response to trauma. Since they tend to intensify the traumatic experience resulting distress, they may be lead to severer post-traumatic stress symptoms [46,47]. Peritraumatic panic attacks were assessed through the Physical Reaction Subscale (PRS) of the Initial Subjective Reaction Scale [48], developed to assess specific cognitive, emotional, and physiological in the face of a traumatic event. The PRS was translated into Brazilian Portuguese for this study.

### Saliva collection

The Oragene™ DNA Self-Collection Kit will be use in order to collect saliva samples. It is an all-in-one system for the collection, preservation, transportation and purification of DNA from saliva [49]. Oragene™ allows the preservation of saliva for 2 weeks under room temperature and DNA extraction in the same amount of those obtained from equivalent blood samples [50]. Saliva collection is a valid option for population approach [50,51] and it is expected to reduce refuses for DNA donation.

For those aged 60 and over few instruments were added in order to address specific issues related to aging:

*The Geriatric Depression Scale GDS) *is a screening questionnaire for depressive symptoms in elderly people [52].

*Physical Self-maintenance Scale and the Instrumental Activities of Daily Living *scale [53].

Hwalek-sengstock elder abuse screening test (H-S/EAST) H-S/EAST is a screening device to identify elderly people at high risk of maltreatment and neglect [54].

*The General Health Questionnaire *is a questionnaire assessing general medical condition widely used for the assessment of common psychiatric disorders, which has been validated in Brazil [55,56].

Mini mental state examination: MMSE is a brief instrument for dementia screening ^18 ^and has been widely used to assess cognitive impairment [57].

### Procedures

A company specialized in household surveys, the Brazilian Institute of Public Opinion and Statistics was hired to carry out the fieldwork. IBOPE provided the interviewers, the physical structure and logistic support for the training, management and supervision. Every single step of the fieldwork was followed by one of the authors (WSR), who had open access to the team engaged in the project.

*Training: *Two of the authors (MIQ and WR) were responsible for training the fieldwork team. The training course comprised a 30-hour theoretical and practical module, followed by a pilot study. In the pilot study, each interviewer applied 10 supervised interviews. Regular meeting with supervision team were carried out in order to solve doubts and standardise the interview procedures. Additionally, the interviewers were given a standardized operation procedure manual covering all aspects of the fieldwork. An additional training was given to the supervision team.

*Data collection: *the data collection was carried out between June/2007 and January/2008 in Sao Paulo, and from October/2007 and July/2008 in Rio de Janeiro. The interviews were carried out in the participants' dwellings. After signing the informed consent, the interviewees were asked to fulfil all the questionnaires. The PTSD section of the CIDI 2.1 and the trauma-related questionnaires were applied only if the interviewee reported at least one traumatic event.

*Quality control: *the supervision team verified all questionnaires within the same week they had been applied. Therefore, the inconsistencies were corrected either by the interviews or by the supervision team within five days. The supervisors re-interviewed at least 20% of all the participants in order to double-check the accuracy of interviewers' work.

*Saliva collection: *after answering the interview, the participants were asked to provide saliva for the genetic analysis. For each subject a 2 ml sample of passively accumulated saliva in a proper recipient (Oragene™) was be collected.

*Data management: *all data were collected onto paper and data entered onto a specific software developed by the IBOPE's data management team. The data was extracted into SPSS format, and the database cleaning, processing of the CIDI 2.1 algorithms and derived variables were performed.

*DNA extraction: *the DNA extraction will follow the standard procedures as recommended by the company (DA Genotek Inc., Canada). The DNA extracted will be quantified by Fluorskan Ascent equipment (Thermo Electron Corporation), using Picogreen^® ^dsDNA quantification reagent (Cambridge Bioscience, U.K). After quantification DNA will be diluted in buffer Te (0.1 mM EDTA, 10 mM Tris HCL, pH 8.0) till a final concentration of 100 n/μl and then transferred to specific tubes identified (ScreenMates 1.4 mL Storage Tubes – Matrix Technologies Corp Ltd.) and store for posterior analyses.

### Data analyses

The statistical analyses will be carried out using SPSS version 16.0 and STATA version 10.0. Given the multi-stage stratified sample design and the oversampling of the most violent areas, all analyses will be weighted to take account of differing selection probabilities at each stage.

For each site the following analyses will be conducted:

1) Description of participants' characteristics: age, gender, marital status, educational level, living arrangements, availability of children for support.

2) Weighted prevalence of post-traumatic stress disorder, hazardous alcohol use and dependence, and common mental disorders, by age and gender, as well as the exposure to traumatic life experience, will be estimated with 95% confidence intervals

3) Prevalence of PTSD, CMD and alcohol related disorders will be compared between the two cities using multivariable regression adjusting for compositional factors such as age, gender, and education. In addition to facilitate comparison with published data we will present standardised prevalences referred to the Brazilian population.

4) The association between traumatic life experience and mental disorders will be estimated adjusting for potential confounders. Multilevel analysis will be performed in order to assess the effect of contextual variables (district homicide rates, the Gini index) upon the prevalence rates.

### Genetic analysis

Genetic analyses will be carried out on DNA of individuals divided according to symptoms counts of General Anxiety Disorder and PTSD as the primary phenotypes and other disorders in following secondary analyses. Whole genome association analysis will be performed on the 1 million SNP (single nucleotide polymorphism) arrays. For loci that are associated at a suggestive level (p < 1 × 10-5) we will carry out association analysis on additional phenotypes. Further analysis will test association of haplotypes in the associated region employing imputation and, if necessary, individual genotyping of proximate common/likely functional SNPs that are unimputable.

### Ethical Issues

Participants were informed about research procedures and risks and signed an informed consent submitted and approved by the Ethical Committee of the Federal University of São Paulo. Subjects who matched diagnostic criteria have been offered a referral to outpatient clinic at the Federal University of Sao Paulo and Federal University of Rio de Janeiro.

## Discussion

Despite the exiting literature, effects of violence on mental health remain under-researched in low and middle-income countries. Several studies have found violence to be associated with PTSD [9,14,17,58], and common mental disorders [59,60]. However, evidence on how this association works is lacking. Moreover, it is still unclear which factors could mediate the association of violence with mental disorders either as risk factors, or protective factors. This study carried out in the two Brazilian biggest cities provides a powerful opportunity for deepening the understanding about the ways violence affects mental health, as well as about the aetiology of PTSD, which is the most frequent psychiatric consequence of exposure to violence.

The study aims to identify factors mediating the association between violence and mental disorders at societal and individual levels, including genetic factors. By doing so, the results may have important clinical and public health implications. Some individual characteristics assessed in the study, such as resilience and positive affects, may help victims of violence to recover from the traumatic experiences, whereas other individual factors such as negative affects and peritraumatic reactions are found to be risk factors for psychopathological outcomes following a traumatic event. Particularly the peritraumatic reactions have been found to be strong predictors of PTSD, as well as of response to treatment.

One could argue that it could be problematic to ask the respondents about their peritraumatic experiences. In fact, trauma experts have raised the concern that research with a focus on psychological trauma could make participants relive auditory or visual reminders of unpleasant situations, which could trigger painful emotions such as fear, anger, and shame. Based on their clinical experience, and on a literature review, the authors found no evidence that asking about peritraumatic reactions is worse than what is usually done when researchers ask about the traumatic memory of the event in an autobiographical perspective. The authors also have noticed that patients usually accept well the peritraumatic questions that index behavioural and physical phenomena such as panic attack and tonic immobility. Patients are usually much more upset when describing the traumatic event than answering if they had tremor, or if they felt paralyzed during an event, or in the aftermath of an event. By assessing these reactions in a community sample, the study may contribute to elucidate some existing controversies about the role of peritraumatic reactions in the course of PTSD.

## Competing interests

The authors declare that they have no competing interests.

## Authors' contributions

SBA, WSR, and MIQ conceived and designed the study. WSR and MIQ were responsible for training the fieldwork team and data collection. WSR wrote the first draft of the Protocol and will be a main investigator as part of his doctorate scholarship and CAPES sandwich scholarship. JJM, MFM, RAB, CPF, CG, GB, SLB, ESFC, MP, TH, MRJ, IF, and RAB have made a substantial contribution to the conception and design of the study and will be supervising data analysis and interpretation of data. CG and GB will be responsible for the genetic study. SLB will be responsible for the geriatric study. MIQ will be responsible for the psychotropic study. LMPV, MSLY, and TSM are post-grad students involved in different parts of the project. AG is a Master of Science student involved in the genetic study. MED, DRL will be participating in the analysis and interpretation of data. LCQ is post-doc student and will be participating of data analysis and interpretation of the results. JJM is head of the main Research Department where the research is conducted and leads this research project. All authors read and approved the final manuscript.

## Pre-publication history

The pre-publication history for this paper can be accessed here:


